# Meningoencephalomyelitis and brachial plexitis in a dog infected with louping ill virus

**DOI:** 10.1177/03009858241265035

**Published:** 2024-07-25

**Authors:** Sai Fingerhood, Karen L. Mansfield, Arran J. Folly, Ana Gomez Vitores, Mara Rocchi, Dominic Clarke, Cecilia Gola

**Affiliations:** 1 University of Surrey Veterinary Pathology Centre, Guildford, UK; 2 Animal and Plant Health Agency, New Haw, Surrey, UK; 3 Moredun Research Institute, Penicuik, UK; 4 South Moor Vets, Kingsbridge, UK

**Keywords:** brachial plexitis, brachial neuritis, canine, louping ill virus, meningoencephalitis, meningoencephalomyelitis, myelitis

## Abstract

A foxhound from a hunting kennel in the United Kingdom was euthanized after being hospitalized with progressive neurologic signs, including tremors, seizures, and obtunded mentation. No abnormalities were appreciated on gross *postmortem* examination. Histologically, severe meningoencephalomyelitis and mild neuritis of the brachial plexus were present. Molecular analysis of brain tissue detected louping ill virus. In addition, louping ill virus-specific antigens were detected in neurons within the brainstem, the entire length of the spinal cord, as well as in rare cells in the brachial plexus using immunohistochemistry. The genetic sequence of the virus appears most closely related to a previously detected virus in a dog from a similar geographic location in 2015. This is the first characterization of the inflammatory lesions and viral distribution of louping ill virus in a naturally infected dog within the spinal cord and brachial plexus.

Louping ill virus (LIV) is a zoonotic, tick-borne, neurotropic, single-stranded RNA virus in the family *Flaviviridae*. The virus is endemic in the upland areas of the United Kingdom and Ireland.^17^ Although LIV is most frequently recorded in the United Kingdom, the geographic range appears to be expanding, with recent detections in Scandinavia.^10,13^ Louping ill virus persists in an enzootic cycle driven primarily by *Ixodes ricinus* ticks. Ecologically significant hosts which contribute to viral persistence and transmission include sheep, mountain hares (*Lepus timidus*), and red grouse (*Lagopus lagopus scoticus*).^11,14^ Disease associated with LIV infection has predominantly been reported in sheep^12^ and red grouse.^20^ However, LIV encephalitis has also infrequently been reported in horses,^22^ cattle,^2^ goats,^1^and humans. Clinical presentations varying from influenza-like symptoms to encephalitis and hemorrhagic fever have been described in rare human cases.^7^ Rare cases of canine LIV infection have also been reported.^6,15,16^

In late September 2022, a 3-year-old, intact female, foxhound dog from south Devon (UK) was hospitalized for neurologic signs. Over 3 days, signs progressed from tremors with hypermetric gait and ataxia to lateral recumbency, obtundation, intermittent seizure-like activity, and eventual coma. Treatments included oral administration of clindamycin (300 mg TID), intravenous fluids (0.9% NaCl), and a single dose of intravenous diazepam. Complete blood counts and biochemistry showed abnormalities including significantly elevated C-reactive protein (85.9 mg/L [0.0-10.0]) and elevations in alanine transferase, alkaline phosphatase, and gamma-glutamyl transferase (360 U/L [reference range: 10-125], 245 U/L [23-212], and 55 U/L [0-11], respectively). Serum indirect fluorescent antibody testing for *Neospora caninum* and *Toxoplasma* sp. IgG, performed by Axiom Laboratories (Newton Abbot, Devon), were negative and borderline positive, respectively. Based on the disease progression and lack of response to treatments, humane euthanasia was elected. The cadaver was then submitted for *postmortem* examination.

Over the 4 years prior to the euthanasia of this dog, multiple other pack members had died after exhibiting neurologic signs similar to those described in this case. A brief report of the deaths in this pack of hounds has recently been published.^5^ The diet of the pack included raw beef and meat from fallen sheep stock.

No changes were identified on gross *postmortem* examination. The entire spinal cord, brachial plexi, sciatic nerves, and brain, along with representative sections of other major organs (lung, liver, kidney, heart, spleen, and skeletal muscle) were retained in 10% neutral-buffered formalin, paraffin embedded, cut in 5-μm sections, and stained with hematoxylin and eosin. Brain samples were frozen at −20°C.

Histologically, bilaterally symmetric, lymphocytic meningoencephalomyelitis was present throughout all examined sections of the brain, brainstem (mibrain, pons, medulla oblongata), spinal cord, and spinal nerve roots, extending to the cauda equina (Fig. 1A–D). The inflammation was characterized by frequent expansion of Virchow–Robin space with dense lymphocytic cuffs, scattered glial nodules, and diffuse gliosis. Occasional neuronal necrosis and neuronophagia was present, most frequently within the thoracic and lumbar spinal cord (Fig. 1B) and unilaterally within the abducens nucleus of the brainstem. While lymphocytic perivascular cuffing was equally present in the meninges, white matter, and gray matter, gliosis and glial nodules more severely affected the gray matter. The gray matter of the cerebellum was relatively unaffected, though the overlying meninges and the ventral portions of the white matter demonstrated similar degrees of inflammation as the cerebrum. The brachial plexus contained multifocal, lymphocytic infiltrates that were less dense than those in the spinal cord (Supplemental Figures S1A, B). The sciatic nerve did not have inflammatory infiltrates.

**Figure 1.**
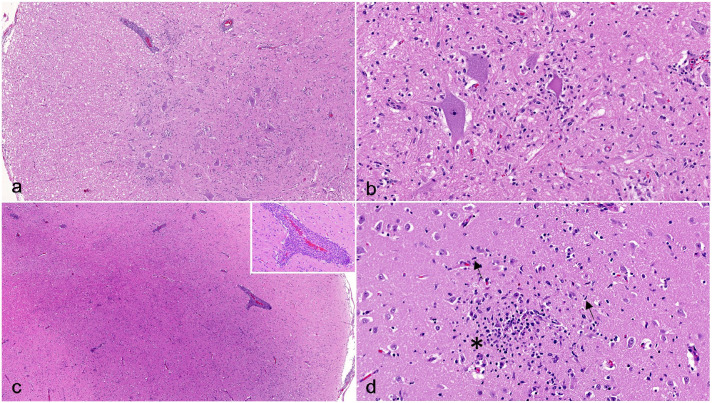
Louping ill virus meningoencephalomyelitis, spinal cord and brain, dog. Hematoxylin and eosin. (a) Lumbar spinal cord. The meninges and perivascular spaces are multifocally expanded with dense clusters of lymphocytes. (b) Lumbar spinal cord gray matter. Necrotic neurons are surrounded by mononuclear inflammatory cells (neuronophagia) and diffuse gliosis. (c) Cerebral cortex. Similar to the spinal cord, the meninges and vessels within the gray and white matter are expanded with dense lymphocytic infiltrates. Inset: lymphocytic perivascular cuffing. (d) Cerebral cortex. Glial nodules are characterized by relatively dense nodular accumulations of microglia mixed with smaller numbers of astrocytes (asterisk). Gliosis is characterized by generalized increased numbers of mixed glial cells, with notably increased numbers of astrocytes and microglia with elongated nuclei (rod cells) (arrows).

Distemper virus quantitative polymerase chain reaction (PCR; University of Bristol, Langford Vets) and *Toxoplasma gondii* immunohistochemistry (IHC; University of Glasgow, Veterinary Diagnostic Services) on brain tissue were negative. Frozen brain tissue was positive for LIV RNA by LIV-specific TaqMan real-time reverse transcription PCR (RT-PCR), a pan-flavivirus real-time RT-PCR, and a LIV/tick-borne encephalitis virus *envelope* gene-specific endpoint RT-PCR (Supplemental Materials). Sanger sequence analysis yielded a 620 bp fragment of the envelope gene, which was submitted to NCBI GenBank (accession number OR759141, Supplemental Table S1). A Bayesian phylogeny indicated that the sequence obtained in this investigation was most closely related (99.83% identity) to a LIV strain detected in 2015 from a dog in Devon, UK^6^ (Supplemental Figure S2). These two dog sequences were grouped with other LIV sequences from the United Kingdom (England, Scotland) and Ireland (Dublin).

Immunohistochemistry targeting LIV antigen-, CD3 antigen-, CD20 antigen-, and using MAC387-specific antibodies was performed (Supplemental Materials) on cerebral, cerebellar, brainstem, spinal cord, and brachial plexus sections. Cytoplasmic immunoreactivity for LIV was present within neuronal cell bodies and processes in the brainstem, spinal cord, and rare cells in the brachial plexus (Fig. 2). No LIV antigen was identified within the cerebellum or cerebrum. Within the brainstem, immunoreactive neurons were present unilaterally within the dorsal nucleus of the trapezoid body and the abducens nucleus. Within the spinal cord, the highest concentrations of immunoreactive neurons were present within the cervical and lumbar sections. The distribution was unilateral within the dorsal and ventral horns of the cervical sections, and bilateral, but mainly within the ventral (motor) horns and within the ventral nerve roots, in the lumbar sections. In the brachial plexus, rare, scattered nucleated cells with moderate amounts of cytoplasm were immunoreactive (Supplemental Figure S1D), which, based on their location, represent either Schwann cells or peripheral nerve resident macrophages. Most inflammatory cells were CD3 antigen positive (T lymphocytes) (Fig. 2, Supplemental Figure S1C). Small numbers of CD20 antigen-positive cells (B lymphocytes) were present within the perivascular cuffs and very rare, individual MAC387-positive cells (macrophages) were present.

**Figure 2.**
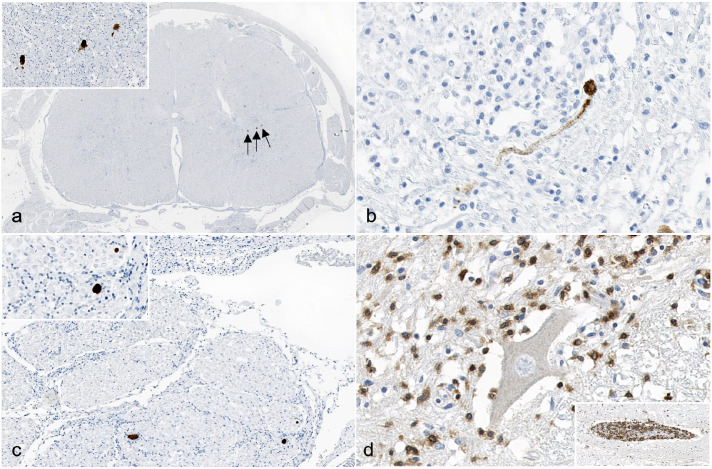
Louping ill virus (LIV) meningoencephalomyelitis, lumbar spinal cord, dog. Immunohistochemistry (IHC) (a-c). Immunolabeling for LIV antigen (arrows) is present in the lumbar spinal cord in (a, inset) neuronal cell bodies of the gray matter, (b) axons within the gray matter, and (c, inset) axons in the ventral spinal nerve roots. LIV IHC. (d) The majority of the inflammatory cells within the gray matter demonstrate strong, cytoplasmic and membranous, granular, CD3 immunolabeling. Inset: Perivascular cuffing with CD3-positive lymphocytes (T lymphocytes). CD3 IHC.

The pattern of inflammation and neuronal necrosis within the brainstem, cerebral cortex, and spinal cord in this case is similar to previous observations in LIV-infected lambs and mice.^8,18,21^ The relative lack of inflammatory infiltrates in the cerebellum and florid CD3 antigen-positive lymphocytic inflammation within the cerebrum and brainstem is similar to that described previously in a dog; however, in that case, relatively more perivascular cuffs were described within the gray matter relative to the white matter, while no significant difference in distribution of perivascular cuffs was appreciated in this case.^6^ Phenotypic characterization of the inflammatory cell infiltrate has only been previously reported in lambs with variable results. One study reported a predominance of plasma cells via ultrastructural examination,^9^ while another study reported a predominance of CD3 antigen-positive lymphocytes via IHC.^21^ Consistent with the latter study, this case showed a predominance of CD3-antigen-positive immune cells clustered around vessels, as well as individually scattered throughout the neuroparenchyma. Rare MAC387-positive macrophages were present in the neuroparenchyma, reflecting a minor component of active histiocytic inflammation. A full characterization of the histiocytic component of inflammation was not pursued and may be an area for future investigation.

Viral antigen was confined to neurons within the brainstem and spinal cord and Schwann cells or macrophages within the brachial plexus with no detection in the cerebellum. This contrasts with a previously reported case of canine LIV where rare neurons within the cerebellar peduncles and Purkinje cells contained viral antigen.^6^ Similar to experimental IHC studies in grouse, mice, and lambs, LIV antigen was identified within neuronal cell bodies and processes in this case.^8,20^ However, LIV antigen immunoreactivity and inflammation within the brachial plexus had not been assessed in cases of experimental infection. The presence of LIV antigen within the brachial plexus could indicate replication of the virus within peripheral nerve plexi and/or reflect a potential route of entry into the central nervous system along neural pathways. Experimental studies in mice on the distribution of a closely related flavivirus, tick-borne encephalitis virus, have demonstrated the presence of viral antigen within the enteric nervous system after intravenous and subcutaneous inoculation.^3,19^ The brachial plexitis identified here warrants further investigation into the distribution of LIV in the peripheral nervous system. The unilateral distribution of the virus within neurons of the cervical spinal cord and the brainstem was also an interesting finding that could also support the hypothesis that this virus can translocate along neural pathways.

Experimental LIV infections in sheep have demonstrated variable distributions of histologic lesions depending on the route of inoculation. With subcutaneous inoculation, there is a relative sparing of the cerebrum with more severe inflammatory lesions in the gray matter of the brainstem, cerebellum, and spinal cord.^8,17^ In these animals, the distribution of viral antigen was consistently reported in the brainstem and spinal cord, with variable presence within the cerebellum. Intranasal inoculation of lambs and mice has resulted in relatively severe forebrain lesions that include localization of LIV antigen within neuronal cell processes, leading to the conclusion that one method of viral spread and transport is along neural pathways.^8,20^ Spread along neural pathways may account for the distribution of viral antigen in the brachial plexus, spinal cord, and brainstem in this case.

The previously sequenced canine case of LIV occurred in close geographic proximity to the current case.^6^ The apparent lack of viral evolution in LIV through time is presumably due to low diversity, with limited evidence for positive selection in the LIV genome, and no evidence of recombination.^4^ Analysis using BEAST has shown the molecular clock rate for LIV to be 1.9 × 10^-5^ substitutions/site/year (95% HPD: 5.7 × 10^−6^ − 3.9 × 10^−5^ substitutions/site/year); however, more extensive analysis would be required to validate this analysis.^4^

The susceptibility of dogs to this disease may depend on a combination of host, viral, and environmental factors. The geographic region that this animal inhabited has relatively high rates of LIV diagnosed in sheep. Consequently, sustained LIV transmission is occurring, most likely facilitated by questing ticks.^12^ If LIV infection was the cause of the neurologic signs in the additional dogs from this kennel that had died over the previous 4 years, it may reflect increased exposure to the virus, particularly in rural hunting dogs trained in areas where the virus is endemic. Recent reports of an increased incidence of fatal LIV infections in hunting dogs within the United Kingdom support this hypothesis.^5^ To our knowledge, this is the first immunolocalization of LIV antigen within the spinal cord of a dog as well as the first immunophenotyping and report of brachial plexitis associated with LIV infection in any species. Although the incidence of confirmed canine cases is rare, our results suggest that LIV should be considered as a differential diagnosis for meningoencephalomyelitis and neuritis/plexitis in dogs within the United Kingdom and regions where LIV is known to circulate.

## Supplemental Material

sj-pdf-1-vet-10.1177_03009858241265035 – Supplemental material for Meningoencephalomyelitis and brachial plexitis in a dog infected with louping ill virus Supplemental material, sj-pdf-1-vet-10.1177_03009858241265035 for Meningoencephalomyelitis and brachial plexitis in a dog infected with louping ill virus by Sai Fingerhood, Karen L. Mansfield, Arran J. Folly, Ana Gomez Vitores, Mara Rocchi, Dominic Clarke and Cecilia Gola in Veterinary Pathology
