# Supplementary material for: Meningoencephalomyelitis and brachial plexitis in a dog infected with louping ill virus
Source: Vet Pathol. 2024 Jul 25;62(1):87–91. doi: 10.1177/03009858241265035 (PMC11697496; doi:10.1177/03009858241265035)
Supplement: sj-pdf-1-vet-10.1177_03009858241265035 – Supplemental material for Meningoencephalomyelitis and brachial plexitis in a dog infected with louping ill virus [file sj-pdf-1-vet-10.1177_03009858241265035.pdf]

Supplemental Materials

Meningoencephalomyelitis and brachial plexitis in a dog with louping ill virus

Sai Fingerhood, Karen L Mansfield, Arran J Folly, Ana Gomez Vitores, Mara Rocchi, Dominic Clarke, Cecilia Gola

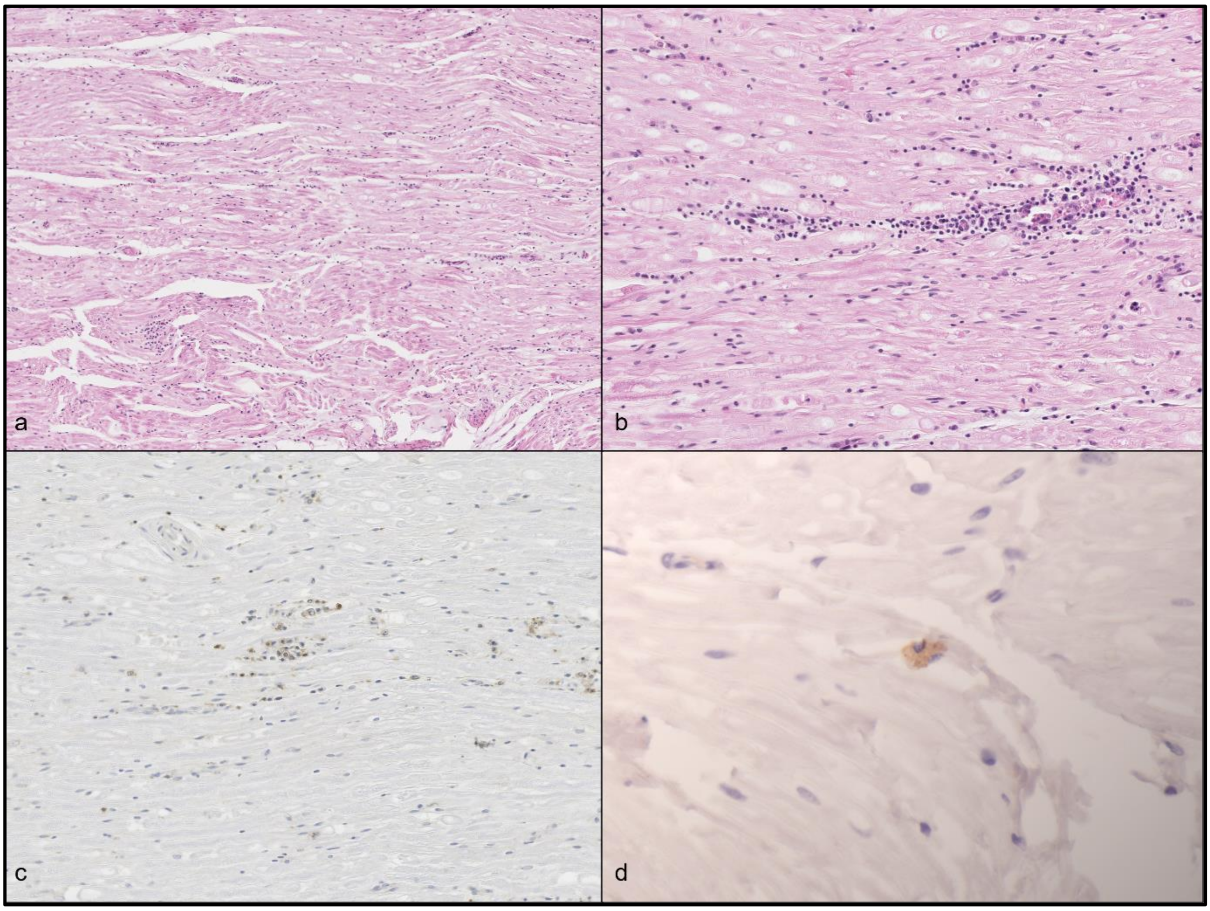

Supplemental Figure S1. Lymphocytic brachial plexitis in a dog with louping ill virus, brachial plexus. **(a-b)** Lymphocytic brachial plexitis. Moderate numbers of lymphocytes multifocally surround blood vessels and percolate between axons. Hematoxylin and eosin. **(c)** CD3 antigen IHC. Lymphocytes demonstrate strong, granular, cytoplasmic immunoreactivity. **(d)** LIV antigen IHC. Multifocally, rare, nucleated cells demonstrate moderate granular, cytoplasmic immunoreactivity.

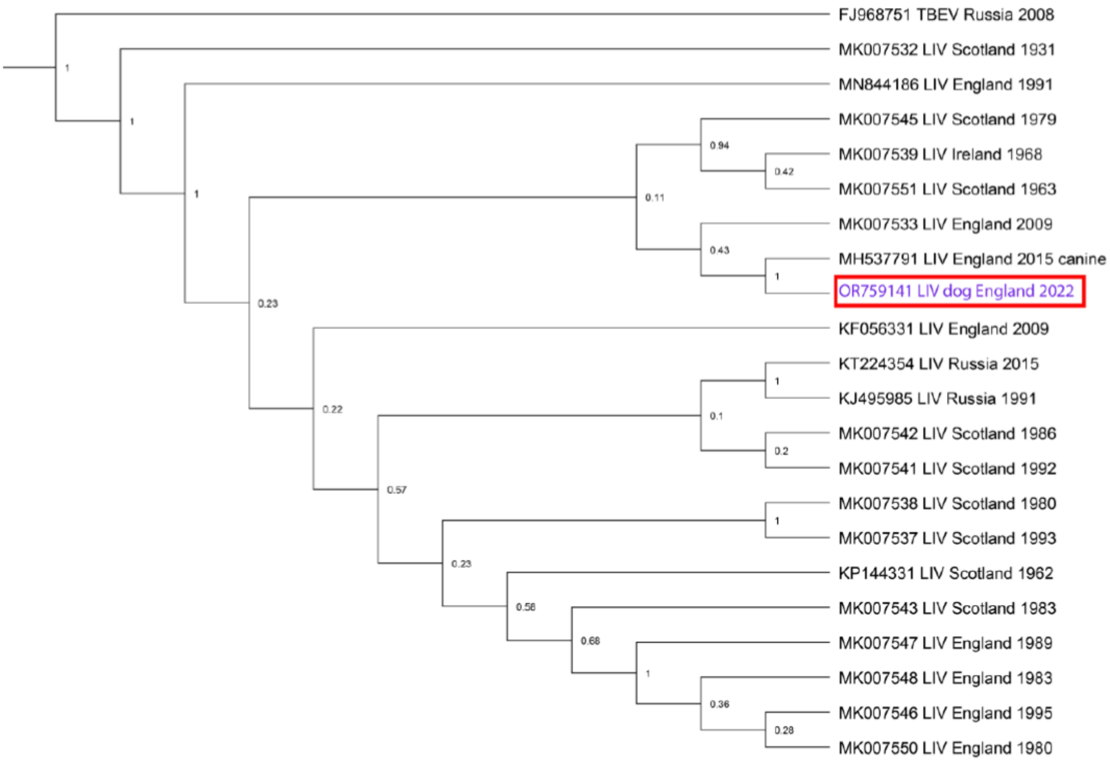

Supplemental Figure S2. Bayesian phylogenetic analysis of louping ill virus (LIV) *envelope* gene sequences, including a 620 bp fragment recovered in this investigation (OR759141). Tick-borne encephalitis virus (TBEV), (Russia 2008, FJ968751) due to its close genetic relationship with LIV, has been included as an outgroup. Our recovered LIV sequenced (purple) was most closely related to a sequence isolated from a dog from Devon in 2015 (sequences are 99.83% identical). Node labels represent posterior probability.

Supplemental Table S1: Louping ill virus (LIV) sequences used for phylogenetic analysis, downloaded from NCBI GenBank or generated in this study (highlighted in bold).

Sequence Name	GenBank Accession	Species	Country	Date
**LIV dog England**	**OR759141**	***Canis lupus familiaris***	**England**	**2022**
SCO_31_1931	MK007532	*Ovis aries*	Scotland	1931
PRES1	MN844186	*Ovis aries*	England	1991
SCO_G_1979	MK007545	*Sus scrofa domesticus*	Scotland	1979
IRE_IRE3_1968	MK007539	*Ovis aries*	Ireland	1968
SCO_369/T2_1963	MK007551	*Ixodes ricinus*	Scotland	1963
ENG_PEN6_2009	MK007533	*Ovis aries*	England	2009
LIV/Dog	MH537791	*Canis lupus familiaries*	England	2015
Penrith	KF056331	*Ovis aries*	England	2009
LEIV-7435Tur	KT224354	*Hyalomma marginatum*	Russia	2015
Primorye-185-91	KJ495985	*Homo sapiens*	Russia	1991
SCO_INV6_1986	MK007542	*Ovis aries*	Scotland	1986
SCO_INV14_1992	MK007541	*Ovis aries*	Scotland	1992
SCO_K_1980	MK007538	*Lagopus lagopus scotica*	Scotland	1980
SCO_LOCH2_1993	MK007537	*Lagopus lagopus scotica*	Scotland	1993
LI3/1	KP144331	*Ovis aries*	Scotland	1962
SCO_INV1_1983	MK007543	*Ovis aries*	Scotland	1983
ENG_DEV2_1989	MK007547	*Ovis aries*	England	1989
ENG_DEV1_1983	MK007548	*Ovis aries*	England	1983
ENG_DEV4_1995	MK007546	*Ovis aries*	England	1985
ENG_A_1980	MK007550	*Ovis aries*	England	1980
Kolarovo-2008[a]	FJ968751	*Ixodes pavlovskyi*	Russia	2008

[a]Tick-borne encephalitis virus sequence used to root the phylogenetic tree.

Supplemental Methods

a. Immunohistochemistry (IHC) methods

Antibody	Supplier	Product code	Clonality	Isotype	Epitope unmasking	Dilution	Incubation time
LIV	Moredun Research Institute	LM3.3D	Monoclonal	Mouse	HIER pH9	1:500	2h at 23ºC
CD3	Dako	A0452	Polyclonal	Rabbit	TBST	1:300	Overnight 4ºC
CD20	Invitrogen	PA5-16701	Polyclonal	Rabbit	T/C	1:500	Overnight 4ºC
MAC387	Biorad	MCA874G	Monoclonal	Mouse	T/C	1:400	1h at 23ºC

Abbreviations: HIER, heat-induced epitope tetrieval; LIV, louping ill virus; TBST, Tris-buffered saline, 0.05% Tween20; T/C, Tris-citrate

For the LIV antigen-specific antibody, a known PCR and IHC positive sheep brain was used as a positive control and a known PCR and IHC negative dog brain was used as a negative control. For the CD3, CD20, and MAC387 antibodies, a multispecies control block containing normal mouse, alpaca, porcine, ovine, and bovine lymph nodes was used as a positive control and included internal negative controls. Additionally, to ensure specificity, the protocol was repeated on all case tissues and positive control blocks using isotype matched antibodies (normal rabbit IgG and normal mouse IgG1) at dilutions matching those of the antibodies of interest, serving as additional negative controls. Negative internal controls were additionally identified within all the case tissues examined.

b. Louping ill virus (LIV) real-time, reverse transcription PCR (RT-PCR) methods

For molecular analysis, RNA was extracted from brain tissue using TRIzol (Invitrogen) according to the manufacturer's instructions. Extracted RNA was assessed using previously described LIV-specific TaqMan RT-PCR,[3] a pan-flavivirus RT-PCR,[1] and an LIV/tick-borne encephalitis virus *envelope* gene-specific endpoint RT-PCR.[2] For all three RT-PCRs, nuclease-free water was used as a negative control, and a known LIV-positive RNA sample was used as a positive control. The endpoint RT-PCR yielded a 620 bp fragment of the envelope gene, which underwent Sanger sequencing, and sequence data was submitted to the NCBI GenBank database under accession number OR759141. For phylogenetic analysis existing LIV isolates available on Genbank and our recovered sequence (OR759141) were aligned using MEGA X. The alignment file was then imported into BEAST v1.10.4 and using the GTR nucleotide substitution model and 10,000,000 Markov chain Monte Carlo generations a Baysian phylogenetic tree was created. Log files were analysed in Tracer v1.7.1 to check effective sample size and a 10% burn-in was included (TreeAnnotator v1.10.4), before tree visualisation and annotation in FigTree v1.4.4.

References

1. Johnson N, Wakeley PR, Mansfield KL, et al. Assessment of a novel real-time pan-flavivirus RT-polymerase chain reaction. *Vector Borne Zoonotic Dis Larchmt N*. 2010;10(7):665–671.

2. Mansfield KL, Johnson N, Banyard AC, et al. Innate and adaptive immune responses to tick-borne flavivirus infection in sheep. *Vet Microbiol*. 2016;185:20–28.

3. Marriott L, Willoughby K, Chianini F, et al. Detection of Louping ill virus in clinical specimens from mammals and birds using TaqMan RT-PCR. *J Virol Methods*. 2006;137(1):21–28.
